# Left Atrial and Right Atrial Deformation in Patients with Coronary Artery Disease: A Velocity Vector Imaging-Based Study

**DOI:** 10.1371/journal.pone.0051204

**Published:** 2012-12-04

**Authors:** Ping Yan, Bin Sun, Haiming Shi, Wen Zhu, Qing Zhou, Yuwen Jiang, Hui Zhu, Guoqian Huang

**Affiliations:** Department of Cardiology, Huashan Hospital, Fudan University, Shanghai, PR China; University of Virginia Health System, United States of America

## Abstract

**Background:**

Impaired left ventricular (LV) function has been shown by strain rate (SR) imaging in patients with coronary artery disease (CAD). Our aim was to investigate global and regional, systolic and diastolic left atrial (LA) and right atrial (RA) longitudinal deformation in CAD using velocity vector imaging.

**Methods:**

Echocardiographic and velocity vector imaging studies were performed in 20 patients with mild CAD, 40 patients with severe CAD and 25 controls. Maximal atrial volume, peak atrial longitudinal strain (ε_s_) and SR during LV systole (SRs), SR during early LV filling (SRe) and late LV filling (SRa) were measured. Longitudinal strain during atrial contraction (ε_a_) was obtained at the onset of P-wave on electrocardiography, and ε_a_/ε_s_ was calculated.

**Results:**

Longitudinal peak ε_s_ and SRs of LA showed decreased trend among CAD patients. The global and lateral LA SRe were prominently lower, while RA ε_a_, SRa and ε_a_/ε_s_ were prominently higher in 2 CAD groups than control group (*P* value <0.05). As compared with controls and patients with other single-vessel disease, LA SRa and ε_a_/ε_s_ ratio were significantly increased among patients with exclusively left anterior descending coronary artery (LAD) stenosis (SRa 1.14±0.38 s^−1^, 1.10±0.41 s^−1^, 1.45±0.46 s^−1^, *P* value<0.05; ε_a_/ε_s_ 0.44±0.11, 0.44±0.20, 0.57±0.12, *P* value<0.01).

**Conclusions:**

Apparently decreased SRe of LA and increased ε_a_, SRa and ε_a_/ε_s_ of RA were found in CAD patients with preserved LVEF and E/E' in gray zone. SRa and ε_a_/ε_s_ of LA were found to significantly increase in those with LAD stenosis.

## Introduction

Coronary artery disease (CAD) is a major public health problem with high risk of developing heart failure. Left ventricular (LV) diastolic dysfunction is often present in patients with significant CAD, even preceding regional or global LV systolic dysfunction, which therefore might serve as an early and sensitive marker of ischemia [1], [2]. Furthermore, it is well known that the atrial contribution is of particular importance in the setting of LV dysfunction to maintain adequate LV stroke volume [3]. Evaluation of LA function may emerge as an important component in assessing the hemodynamic effects of many diseases.

In recent years, accumulative evidence has shown that strain (ε) and strain rate (SR) are powerful echocardiographic parameters to directly reflect global and regional systolic and diastolic myocardial deformation [4]–[8], and to sensitively detect dysfunction from myocardial ischemia in CAD patients [9]–[12]. The measurement of atrial deformation by strain method is a promising and useful tool, but there are few data on the ischemia-related alterations of atrial myocardial deformation. The aim of this study is to evaluate the function of both atrial myocardium in CAD patient using vector velocity imaging (VVI), and also to test our novel hypothesis that atrial impairment might be associated with the severity of coronary stenosis and the distribution pattern of obstructive coronary artery.

## Methods

### Study Participants

Patients with suspected CAD and undergone coronary angiography in Huashan Hospital between May 2009 and January 2010 were continuously enrolled. To minimize the influence of some serious or complex medical conditions, we excluded patients with acute myocardial infarction or history of coronary revascularization (including coronary artery bypass grafting and percutaneous coronary intervention), diabetes mellitus, New York Heart Association (NYHA) class III–IV heart failure, abnormal cardiac rhythm (other than sinus rhythm). Also excluded were those with suboptimal angiographical or echocardiographic images. As a result, 85 participants were included in the final analysis. The study was approved by the medical ethics review committee of the Huashan Hospital and all subjects gave written informed consent.

Demographic data and cardiovascular risk factors were collected, including age, gender, weight, height, systolic blood pressure (SBP), diastolic blood pressure (DBP), and the history of hypertension, status of smoking. Body mass index (BMI) was calculated as weight in kg divided by height in meters squared [13]. Blood samples were collected after 12-hour overnight fasting. All samples were analyzed for serum total cholesterol, high-density lipoprotein cholesterol (HDL-C), low-density lipoprotein cholesterol (LDL-C), triglycerides, and blood glucose by enzymatic methods with an automatic analyzer (Hitachi 7600–020 Automated Analyzer; Hitachi, Tokyo, Japan). Results of a 75 g oral glucose tolerance test (OGTT) were recorded as well. Day-to-day coefficients of variation for all analyses were 1% to 2% at the central laboratory in our hospital.

Standard selective coronary angiography was performed using the Judkins technique, by a qualified catheterizing cardiologist and an angiographer who were blinded to the study. CAD was defined as ≥50% lumen narrowing of at least one major coronary vessel [14], [15]. And the percentage of stenosis in each main branch was documented. Accordingly, the participants were classified into the following three categories: 1) control group without CAD; 2) mild CAD group with borderline (50–70%) coronary stenosis; and 3) severe CAD group with coronary stenosis greater than 70%.

### Echocardiography

Transthoracic echocardiography was performed on the subjects at rest in the left lateral decubitus position by 2 professional cardiologists with a Siemens Sequoia 512 ultrasound machine using a 3V2C transthoracic transducer (Siemens Medical Systems, Mountain View, CA, USA), 1–2 days before the angiographic studies. Complete two-dimensional, color, pulsed and continuous-wave doppler examinations were performed according to standard techniques [16], [17]. Parasternal long-axis views were used to derive the M-Mode measurements of LA size, LV end-diastolic interventricular septal (IVST) and posterior wall thickness (PWT), and LV end-diastolic (LVDd) and end-systolic dimensions (LVDs). LV mass (LVM) was calculated using the regression equation described by Devereux et al [18], i.e. LVM  = 1.04×((IVST + PWT + LVDd) ^3^– LVDd ^3^) –13.6, and was corrected to body surface area [19]. LV fractional shortening (LVFS) was calculated as (LVDd – LVDs)/LVDd. LV ejection fraction (LVEF) was calculated by the modified biplane Simpson rule and expressed as a percentage. From the LV inflow spectrum (measured at the tips of the mitral valve), the transmitral peak E-wave velocity, E wave deceleration time and peak A-wave velocity were recorded during quiet breathing. The ratio of maximal mitral flow velocities (E/A ratio) was calculated. In addition, the septal mitral annulus early (E') velocity was measured by tissue doppler imaging, and the E/E' ratio was calculated using a cutoff value >15 to represent elevated LV filling pressure [20]. All echocardiographic measurements used in the analysis were averaged from 3 heart beats [5].

### VVI Analysis

For the assessment of longitudinal atrial deformation, two-dimensional grey-scale image of apical 4-chamber view was obtained under VVI mode with highest possible frame rate and a stable electrocardiogram recording. Special attention was paid to avoid foreshortening the atrium and to gain a reliable delineation of the atrial endocardial border. Cine loops with 2–3 consecutive heart cycles during breath hold were acquired and saved digitally.

**Figure 1:**
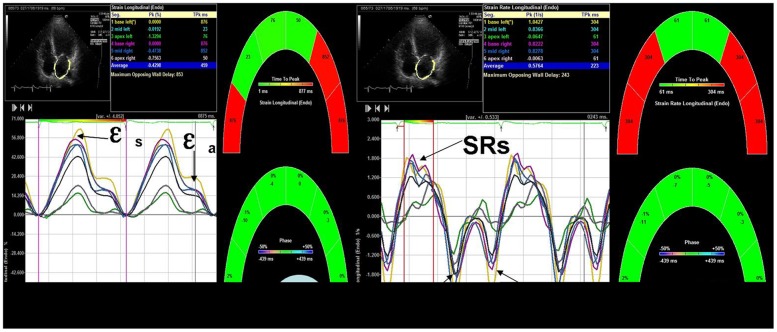
Left atrial longitudinal strain/strain rate curves obtained from an apical four-chamber view. ε_s_: peak atrial longitudinal strain during ventricular systole, ε_a_: atrial strain at the onset of P-wave on electrocardiography, SRs: peak left atrial strain during LV systole, SRe: peak left atrial strain during early LV diastole, SRa: peak left atrial strain during late LV diastole.

Strain analysis of LA and RA was performed offline with Siemens syngo US workplace (version 2, Siemens Medical Solutions USA). After manually defining the endocardial border of both atriums, the myocardial speckle was automatically tracked frame-by-frame by the VVI software throughout the cardiac cycle to calculate and generate strain/strain rate curves. Besides LA and RA global longitudinal function, regional atrial longitudinal strain/strain rates of the interatrial septum and lateral wall were also evaluated respectively. As shown in Figure 1, peak atrial longitudinal strain (ε_s_) and peak strain rate (SRs) were measured at LV systolic phase, while peak atrial longitudinal SRe during early LV filling and SRa during late LV diastolic phase were measured. Longitudinal strain during atrial contraction (ε_a_) was obtained at the onset of the P-wave on electrocardiography, and the ε_a_/ε_s_ ratio was calculated (corresponding to the contribution of atrial active contraction to the whole atrial deformation during a cardiac cycle). Additionally, atrial time-volume curve and dV/dt curve were rendered automatically by VVI software. Maximal atrial volume and peak atrial dV/dt at ventricular systole were determined.

Interobserver and intraobserver variability for atrial strain/strain rate were examined in an analysis of 20 randomly selected patients. Measurements were performed by one observer, and then repeated two separate times by two observers who were unaware of the other's measurements. More than 4 weeks elapsed between the two readings by the same observer with blinding to the initial measurements.

### Statistical Analysis

Statistical analysis was performed using SPSS 15.0 statistical software (SPSS Inc., Chicago, Ill., USA). Continuous data were expressed as means ± SD, and categories data as percentages. Continuous variables were compared using Student's t-test, or ANOVA when appropriate. Furthermore, Pearson's and Spearman's (for nonnormally distributed data) coefficients of correlation were used where appropriate. All of the reported P values were two-sided with statistical significance evaluated at 0.05.

## Results

### Clinical Characteristics

The clinical data of the 85 participants are presented in Table 1. There was no difference in age, gender distribution, blood pressure, blood glucose/NT-proBNP levels, or kidney function among the 3 groups. None was found to have plasma NT-proBNP >200 pg/ml. Blood lipid levels between groups were also similar, except that triglycerides in patients with severe CAD were higher. The proportions of hypertensive subjects were 15% in mild CAD group, 22% in severe CAD group, and 20% in control group (*P* value, 0.66). There was no difference in history of medical therapy between the 3 groups.

**Table 1:** Baseline clinical data.

Variable	control group	mild CAD group	severe CAD group	*P* Value
	(n = 25)	(n = 20)	(n = 40)	
Age (y)	60.56±8.89	67.60±8.57	62.03±12.39	0.07
Male gender	17 (68%)	14 (70%)	25 (62.5%)	0.82
BMI (kg/m^2^)	23.54±7.05	25.39±4.96	23.29±4.45	0.58
Heart rate (min^−1^)	73.45±8.32	72.25±15.07	69.56±9.89	0.16
SBP (mmHg)	126.23±14.2	125.52±17.62	133.37±18.05	0.22
DBP (mmHg)	77.44±10.23	73.66±10.52	78.13±9.24	0.33
eGFR^&^ (ml/min/1.73 m^2^)	82.70±16.67	83.89±15.08	77.03±22.59	0.82
FPG (mmol/L)	5.79±1.33	4.97±0.82	6.72±2.45	0.57
2-h oral glucose (mmol/L)	8.10±3.17	6.83±2.96	7.74±3.19	0.63
HbA1c (%)	6.32±0.90	5.48±1.09	5.65±1.89	0.29
Cholesterol (mmol/L)	4.31±0.94	4.14±1.1941	5.03±1.41	0.10
Triglyceride (mmol/L)	2.09±1.05	2.51±1.52	3.17±2.22*	0.04
LDL-C (mmol/L)	2.62±0.91	2.45±0.81	2.90±1.34	0.56
HDL-C (mmol/L)	1.01±0.24	1.17±0.36	0.99±0.27	0.31
NT-proBNP (pg/ml)	86.01±23.21	97.23±27.26	115.07±36.34	0.56
hs-CRP(mg/L)	2.19±2.97	2.36±3.23	2.32±2.99	0.98
Current Smoking	7 (28%)	5 (25%)	15 (37.5%)	0.12
HT	5 (20%)	3 (15%)	10 (22.2%)	0.66
Medications				
ACEI and/or ARB	14 (56%)	8 (40%)	20 (50%)	0.56
Beta-blocker	7 (28%)	8 (40%)	13 (32.5%)	0.69
CCB	5 (20%)	4 (20%)	13 (32.5%)	0.42

Abbreviations: BMI, body mass index; SBP, systolic blood pressure; DBP, diastolic blood pressure; FPG, fasting plasma glucose; HbA1c, glycated hemoglobin A1c; LDL-C, low-density lipoprotein cholesterol; HDL-C, high density lipoprotein cholesterol; NT-proBNP, N-terminal pro-brain natriuretic peptide; hs-CRP, high sensitivity C-reactive protein; HT, hypertension; ACEI, angiotensin-converting enzyme inhibitor; ARB, angiotensin II receptor blocker; CCB, calcium channel blocker.

* p<0.05 versus control group.

& Abbreviated MDRD equation: estimated glomerular filtration rate (eGFR), in mL/min per 1.73 m^2^ = 186.3×SCr (exp [−1.154]) × Age (exp[−0.203]) × (0.742 if female) × (1.21 if black).

Of the 60 CAD patients, 17 had exclusively left anterior descending coronary artery (LAD) stenosis, and 10 had exclusively left circumflex coronary artery (LCX) or right coronary artery (RCA) stenosis. 33 had multiple-vessel disease. Of all the patients, 33 were successfully treated by percutaneous coronary intervention with stent implant, while 7 patients needed subsequent coronary arterial bypass grafting surgery.

### Echocardiographic Features

Conventional transthoracic echocardiographic parameters of the study population are presented in Table 2. All the subjects had normal LV diastolic and systolic dimensions. 2 patients with severe CAD were found with mild decreased LV systolic function (LVEF 45–50%). However, there was no significant difference in LVEF or LVFS between 3 groups. The LA dimensions in control, mild CAD and severe CAD groups were 36.36±4.07 mm, 38.50±4.15 mm, 36.68±4.74 mm, respectively (*P* Value, 0.22). Compared with control group, the 2 CAD groups had lower E/A ratio and higher E/E' ratio, but the differences didn't reach statistical significance. None were found to have E/E' ratio >15. Five (8.3%) patients with CAD were found with mild mitral regurgitation, whereas none had moderate to severe regurgitation.

**Table 2:** Echocardiographic parameters in patients and controls.

Variables	control group	mild CAD group	severe CAD group	P Value
	(n = 25)	(n = 20)	(n = 40)	
Ao (mm)	33.32±3.59	32.50±3.69	31.97±2.93	0.29
LA (mm)	36.36±4.07	38.50±4.15	36.68±4.74	0.22
LVDd (mm)	48.72±3.77	49.05±5.61	47.18±3.98	0.20
LVDs (mm)	30.72±3.87	30.55±4.92	29.33±3.12	0.29
IVST (mm)	9.68±1.37	10.05±1.93	10.03±1.56	0.65
LVPWT (mm)	9.20±1.00	9.30±1.26	9.40±1.53	0.84
SV (mL)	85.72±17.52	90.46±29.41	79.88±21.99	0.22
CI (L/min/m^2^)	2.93±0.70	3.24±1.10	2.74±0.90	0.13
LVFS (%)	37.03±5.08	37.85±4.60	37.80±4.42	0.78
LVEF (%)	66.38±6.34	65.30±11.16	66.61±6.39	0.82
E velocity (cm/s)	77.80±14.73	69.00±21.07	72.85±19.92	0.29
A velocity (cm/s)	78.76±19.23	81.00±18.73	81.15±17.20	0.86
DT (ms)	236.75±35.64	213.72±46.32	200.21±51.26	0.22
E/A	1.05±0.34	0.88±0.31	0.94±0.35	0.24
E/E'	6.62±2.53	8.24±2.17	8.60±2.58	0.21
LVMI (g/m^2^)	93.12±25.81	100.57±35.86	97.89±27.30	0.31

Abbreviations: Ao, aorta; LA, left atrium; LVDd, left ventricular end-diastolic dimension; LVDs, left ventricular end-systolic dimension; IVST, interventricular wall thickness; LVPWT, left ventricular posterior wall thickness; SV, stroke volume; CI, cardiac index; LVFS, left ventricular fractional shortening; LVEF, left ventricular ejection fraction; DT, E-wave deceleration time; LVMI, left ventricular mass index.

### VVI Analysis

Data from VVI by the severity of coronary stenosis are summarised in Table 3. The VVI analysis was feasible among all subjects with acceptable echocardiographic images. The average values for the global maximal LA volume index in control, mild CAD and severe CAD groups were 30.41±11.73 mL/m^2^, 33.68±9.34 mL/m^2^, 31.41±11.21 mL/m^2^ (P Value, 0.60). No differences in the LA and RA Peak dv/dt were observed. Longitudinal ε_s_ and SRs of LA tended to be decreased among CAD patients, even though the differences didn't reach statistical significance. Compared with those in the control group, the 2 CAD groups had lower global and lateral SRe (*P* value <0.05), without significant further decrease with increasing severity of coronary stenosis. LA lateral SRa was increased in severe CAD group. By contrast, SRe of RA didn't differ across 3 groups, whereas RA global and lateral ε_a_, SRa and ε_a_/ε_s_ ratio was apparently increased in mild and severe CAD groups.

**Table 3:** Velocity vector imaging-based measurements of LA/RA according to the severity of coronary stenosis.

Variable	control group	mild CAD group	severe CAD group	P Value Overall
	(n = 25)	(n = 20)	(n = 40)	
LA Global				
maximum volume, cm^3^	62.34±19.78	67.11±15.46	65.5±22.18	0.71
maximal LA volume index, mL/m^2^	30.41±11.73	33.68±9.34	31.41±11.21	0.60
Peak dv/dt, ml/s	151.77±50.05	148.53±41.36	156.12±62.89	0.87
ε_s_, %	32.39±10.31	28.67±9.71	28.69±8.75	0.31
ε_a_,%	17.94±9.99	13.48±4.45	14.59±6.06	0.08
SRs,s^−1^	1.29±0.38	1.15±0.22	1.16±0.29	0.19
SRe,s^−1^	−1.08±0.30	−0.84±0.45*	−0.93±0.34*	0.03
Sra,s^−1^	−1.14±0.38	−1.07±0.36	−1.22±0.48	0.43
ε_a_/ε_s_ ratio	0.46±0.11	0.51±0.15	0.49±0.15	0.43
LA lateral				
ε_s_, %	33.94±10.36	31.58±9.24	29.68±7.67	0.18
ε_a_,%	15.27±5.92	15.23±5.08	13.20±5.72	0.25
SRs,s^−1^	1.54±0.50	1.30±0.37	1.33±0.44	0.14
SRe,s^−1^	−1.21±0.32	−0.88±0.49^**^	−1.01±0.32*	0.01
SRa,s^−1^	−1.07±0.41	−1.17±0.35	−1.45±0.68^**&^	0.02
ε_a_/ε_s_ ratio	0.45±0.09	0.50±0.17	0.44±0.16	0.27
Septum				
ε_s_, %	35.06±14.00	28.38±10.48	32.10±11.84	0.17
ε_a_,%	16.38±7.49	14.25±6.38	17.39±7.89	0.31
SRs,s^−1^	1.23±0.44	1.22±0.37	1.22±0.48	0.99
SRe,s^−1^	−1.05±0.36	−0.89±0.44	−0.88±0.33	0.17
SRa,s^−1^	−1.14±0.38	−1.14±0.44	−1.33±0.59	0.22
ε_a_/ε_s_ ratio	0.47±0.11	0.53±0.19	0.55±0.19	0.19
RA Global				
maximum volume, cm^3^	61.57±20.07	57.04±15.66	52.46±16.43	0.12
maximal RA volume index, mL/m^2^	29.97±10.26	28.46±8.78	25.10±7.87	0.09
Peak dv/dt, ml/s	133.34±43.84	116.74±43.02	117.65±52.02	0.39
ε_s_, %	39.20±14.46	43.78±14.74	39.49±14.74	0.50
ε_a_, %	15.23±7.54	22.16±9.35*	19.36±9.13*	0.04
SRs,s^−1^	1.28±0.43	1.24±0.51	1.23±0.42	0.91
SRe,s^−1^	−0.94±0.39	−0.74±0.35	−0.86±0.35	0.21
SRa,s^−1^	−1.04±0.46	−1.29±0.49	−1.38±0.52^**^	0.03
ε_a_/ε_s_ ratio	0.38±0.15	0.52±0.16^**^	0.52±0.15^**^	0.00
RA lateral				
ε_s_, %	52.85±22.85	59.69±19.89	55.66±25.55	0.64
ε_a_, %	21.58±12.04	31.95±21.61	29.25±15.06	0.07
SRs,s^−1^	1.67±0.71	1.71±0.67	1.63±0.59	0.89
SRe,s^−1^	−1.12±0.45	−1.07±0.51	−1.09±0.44	0.93
SRa,s^−1^	−1.28±0.72	−1.74±0.73*	−1.76±0.79*	0.04
ε_a_/ε_s_ ratio	0.41±0.17	0.54±0.22*	0.54±0.13^**^	0.03

* p<0.05 versus control group;^ **^p<0.01 versus control group; ^&^ p<0.05 versus mild CAD group.

The results of LA deformation analysis by the distribution pattern of involved coronary artery (left anterior descending coronary artery (LAD), left circumflex coronary artery (LCX), and right coronary artery (RCA)) are shown in Table 4. Among the patients with exclusively LAD stenosis and those with exclusively LCX or RCA stenosis, maximal LA volumes remained similar while longitudinal LA global SRe decreased appreciably as compared with the controls. However, SRa and ε_a_/ε_s_ ratio of LA were significantly increased only in LAD stenosis group (*P* value<0.05, 0.01, respectively). No similar patterns were found in global RA deformation properties (data not shown).

**Table 4:** Global deformation analysis of LA by the distribution pattern of obstructive coronary artery.

Variable	control group	LAD group	LCX/RCA group	P Value Overall
	(n = 25)	(n = 17)	(n = 10)	
LA Global				
maximum volume	62.34±19.78	58.09±14.42	67.51±20.70	0.44
Peak dv/dt	151.77±50.05	136.53±46.67	170.27±49.61	0.23
ε_s_, %	39.71±15.84	29.74±9.29*	30.41±11.54	0.04
ε_a_, %	17.94±9.99	16.87±6.91	12.03±3.40	0.16
SRs,s^−1^	1.29±0.38	1.13±0.26	1.28±0.23	0.28
SRe,s^−1^	−1.06±0.32	−0.92±0.42	−0.95±0.46	0.49
SRa,s^−1^	−1.14±0.38	−1.45±0.46* ^#^	−1.10±0.41	0.04
ε_a_/ε_s_ ratio	0.44±0.11	0.57±0.12^ **#^	0.44±0.20	0.01

Abbreviations: LAD, left anterior descending coronary artery; LCX, left circumflex coronary artery; RCA, right coronary artery.

* p<0.05 versus control group; **p<0.01 versus control group; ^#^ p <0.05 versus LCX/RCA group.

Interobserver variability for strain was 5±7%, and intraobserver variability was 1.6±0.6%. In addition, inter- and intraobserver variability for strain rate was 0.09±0.06 s^−1^ and 0.07±0.05 s^−1^, respectively.

## Discussion

The atrium has an important role in optimizing overall cardiac function, acting as a reservoir, a conduit, and a booster pump for blood returning to the heart [21]. The changes in LA size and function are associated with cardiovascular disease and are risk factors for atrial fibrillation, stroke, and death [7], [22]. We evaluated comprehensive atrial functions among CAD patients, and investigated the association between atrial deformation and the severity of CAD or the distribution pattern of involved coronary artery, by using VVI. Our study showed a high feasibility. CAD patients were found with decreased SRe of LA as well as increased ε_a_, SRa and ε_a_/ε_s_ ratio of RA. Patients with exclusively LAD stenosis were found with significantly enhanced SRa and ε_a_/ε_s_ ratio of LA, while patients with exclusively LCX/RCA stenosis were not.

LA function measured as volumetric parameters by real-time three-dimenstional echocardiography is considered a robust marker of LV filling pressure and an indicator of LV diastolic function [23]. Recent studies have shown that LA myocardial deformation parameters might be reduced before the atrial volume was changed [24], [25]. Furthermore, it is recommended to use several, rather than single doppler echocardiographic technique for the accurate assessment of cardiac diastolic function [26]. VVI is an emerging and promising angle-independent echocardiographic technique to measure strain by speckles tracking, which overcomes the limitations of tissue doppler imaging [27]. Numerous studies have demonstrated that strain/strain rate parameters are sensitive descriptors of regional myocardial deformation function in evaluating myocardial ischemia [9], [10], [28], [29], and are of additional benefit to conventional myocardial functional measures [30]. However, most studies focused on LV function. The present study showed changes of artrial strain/strain rate, even in CAD patients with normal LA size, preserved LVEF and equivocal E/E'. These findings indicated that the functional assessments of LA/RA could potentially be useful, and may emerge as an important component in assessing the hemodynamic changes in clinical practice. The ε_a_/ε_s_ ratio may represent a new index of atrial contractile function that deserves further assessment. And future study is warranted to evaluate whether these novel echocardiographic parameters can predict enlargement of LA or development of LV diastolic dysfunction or arrhythmias.

Previous studies have proven that E/E' ratio in gray zone (8 to 15) are limited in the estimation of LV filling pressures [20], [31]. In this case, elevated plasma NT-proBNP level would provide incremental diagnostic evidence [32], [33]. According to the non-invasive assessments, none of the patients in our study were found to have definitely elevated LV filling pressure (E/E' ratio >15, or NT-proBNP >200 pg/ml), that might minimize the effect of elevated LV filling pressure on atrial function. We observed that our patients still had significantly more decreased atrial SRe, which probably indicated impaired myocardial dysfunction of LA. Moreover, we found that SRa and ε_a_/ε_s_ ratio of LA was significantly enhanced in patients with LAD stenosis. One explanation could be that hyperactive LA booster pump action compensated for the diminution of LV stroke work [34], [35], whilst no similar founding was shown in patients with LCX/RCA stenosis, possibly due to atrial ischemia caused by obstructive LCX/RCA branches that supply the atrium [36], [37]. However, it can still be discussed that increased SRa and ε_a_/ε_s_ ratio of LA could be due to altered left ventricular compliance with shifting of left ventricular filling to late systole. It is somewhat unexpected that we did not observe a significant difference in the LA/RA deformation parameters between severe coronary stenosis and mild stenosis groups. The exact explanation was unclear. Further studies are necessary to investigate these issues and clarify the detailed mechanisms.

### Study Limitations

The quality of speckle tracking depends highly on the spatial resolution of the image and on the frame rate of the cine-loop. Without dedicated software, measuring strain and strain rate in the atrium is challenging, and can be influenced by nonatrial physiological factors including LV compliance and mitral annular descent. However, recent work [38], [39], including the present study, has shown that direct measurement of atrial deformation using speckle tracking method is feasible and reproducible, and can be used to evaluate LA function. The region of interest for VVI has no width for longitudinal strain/strain rate measurement. Therefore in this regard, VVI may be well-suited to study the deformation of atriums with smooth surface and thin wall, as compared with other speckle tracking software. Our results might add insight to the understanding of atrial mechanics, even before its enlargement. Neverthless, our study had limited power due to the small sample, and the results couldn't be generalized to wider population. Left ventricular filling pressure was not measured directly in the catheterization laboratory. Evaluation of the coronary artery anatomy didn't include a detailed assessment of coronary artery branches that supply the atriums. And long-term clinical outcome data, such as echocardiographic follow-up, cardiovascular event rates and survival assessment were not part of the present study. Further studies are necessary to investigate these issues.

### Conclusions

CAD patients with normal LA size, preserved EF and E/E' in gray zone showed decreased SRe of LA and increased εa, SRa and ε_a_/ε_s_ ratio of RA. SRa and ε_a_/ε_s_ of LA was found to increase in those with LAD stenosis. Further profound studies are warranted to confirm the present findings and define the cut-off values as well as technical standards.

## References

[1] Bonow RO, Bacharach SL, Green MV, Kent KM, Rosing DR, et al (1981) Impaired left ventricular diastolic filling in patients with coronary artery disease: assessment with radionuclide angiography. Circulation 64: 315–323. 7249299 10.1161/01.cir.64.2.315

[2] Lee KW, Blann AD, Lip GY (2005) Impaired tissue Doppler diastolic function in patients with coronary artery disease: relationship to endothelial damage/dysfunction and platelet activation. Am Heart J 150: 756–766. 16209979 10.1016/j.ahj.2004.11.019

[3] Leung DY, Boyd A, Ng AA, Chi C, Thomas L (2008) Echocardiographic evaluation of left atrial size and function: current understanding, pathophysiologic correlates, and prognostic implications. Am Heart J 156: 1056–1064. 19032999 10.1016/j.ahj.2008.07.021

[4] Tanaka H, Kawai H, Tatsumi K, Kataoka T, Onishi T, et al (2007) Relationship between regional and global left ventricular systolic and diastolic function in patients with coronary artery disease assessed by strain rate imaging. Circ J 71: 517–523. 17384452 10.1253/circj.71.517

[5] Yan P, Li H, Hao C, Shi H, Gu Y, et al (2010) 2D-Speckle Tracking Echocardiography Contributes to Early Identification of Impaired Left Ventricular Myocardial Function in Patients with Chronic Kidney Disease. Nephron Clin Pract 118: c232–c240. 21196768 10.1159/000321383

[6] Leung DY, Ng AC (2010) Emerging clinical role of strain imaging in echocardiography. Heart Lung Circ 19: 161–174. 20149727 10.1016/j.hlc.2009.11.006

[7] Kim DG, Lee KJ, Lee S, Jeong SY, Lee YS, et al (2009) Feasibility of two-dimensional global longitudinal strain and strain rate imaging for the assessment of left atrial function: a study in subjects with a low probability of cardiovascular disease and normal exercise capacity. Echocardiography 26: 1179–1187. 19725856 10.1111/j.1540-8175.2009.00955.x

[8] Vianna-Pinton R, Moreno CA, Baxter CM, Lee KS, Tsang TS, et al (2009) Two-dimensional speckle-tracking echocardiography of the left atrium: feasibility and regional contraction and relaxation differences in normal subjects. J Am Soc Echocardiogr 22: 299–305. 19258177 10.1016/j.echo.2008.12.017

[9] Perk G, Kronzon I (2009) Non-Doppler two dimensional strain imaging for evaluation of coronary artery disease. Echocardiography 26: 299–306. 19291015 10.1111/j.1540-8175.2008.00863.x

[10] Tsai WC, Liu YW, Huang YY, Lin CC, Lee CH, et al (2010) Diagnostic value of segmental longitudinal strain by automated function imaging in coronary artery disease without left ventricular dysfunction. J Am Soc Echocardiogr 23: 1183–1189. 20833507 10.1016/j.echo.2010.08.011

[11] Hoit BD (2011) Strain and strain rate echocardiography and coronary artery disease. Circ Cardiovasc Imaging 4: 179–190. 21406664 10.1161/CIRCIMAGING.110.959817

[12] Dattilo G, Lamari A, Zito C, Carerj S, Marte F, et al (2010) 2-Dimensional Strain echocardiography and early detection of myocardial ischemia. Int J Cardiol 145: e6–e8. 19185369 10.1016/j.ijcard.2008.12.100

[13] Keys A, Fidanza F, Karvonen MJ, Kimura N, Taylor HL (1972) Indices of relative weight and obesity. J Chronic Dis 25: 329–343. 4650929 10.1016/0021-9681(72)90027-6

[14] Gurbel PA, Kreutz RP, Bliden KP, DiChiara J, Tantry US (2008) Biomarker analysis by fluorokine multianalyte profiling distinguishes patients requiring intervention from patients with long-term quiescent coronary artery disease: a potential approach to identify atherosclerotic disease progression. Am Heart J 155: 56–61. 18082490 10.1016/j.ahj.2007.08.021

[15] Sun T, Huang Y, Phillips MI, Luo X, Zhu J, et al (2010) Growth differentiation factor 15 and coronary collateral formation. Clin Cardiol 33: E1–E5. 10.1002/clc.20698 PMC6653245 20014173

[16] Lang RM, Bierig M, Devereux RB, Flachskampf FA, Foster E, et al (2005) Recommendations for chamber quantification: a report from the American Society of Echocardiography's Guidelines and Standards Committee and the Chamber Quantification Writing Group, developed in conjunction with the European Association of Echocardiography, a branch of the European Society of Cardiology. J Am Soc Echocardiogr 18: 1440–1463. 16376782 10.1016/j.echo.2005.10.005

[17] Nishimura RA, Miller FA Jr, Callahan MJ, Benassi RC, Seward JB, et al (1985) Doppler echocardiography: theory, instrumentation, technique, and application. Mayo Clin Proc 60: 321–343. 3887049 10.1016/s0025-6196(12)60540-0

[18] Devereux RB, Alonso DR, Lutas EM, Gottlieb GJ, Campo E, et al (1986) Echocardiographic assessment of left ventricular hypertrophy: comparison to necropsy findings. Am J Cardiol 57: 450–458. 2936235 10.1016/0002-9149(86)90771-x

[19] Mosteller RD (1987) Simplified calculation of body-surface area. N Engl J Med 317: 1098. 3657876 10.1056/NEJM198710223171717

[20] Ommen SR, Nishimura RA, Appleton CP, Miller FA, Oh JK, et al (2000) Clinical utility of Doppler echocardiography and tissue Doppler imaging in the estimation of left ventricular filling pressures: A comparative simultaneous Doppler-catheterization study. Circulation 102: 1788–1794. 11023933 10.1161/01.cir.102.15.1788

[21] Mitchell JH, Gilmore JP, Sarnoff SJ (1962) The transport function of the atrium. Factors influencing the relation between mean left atrial pressure and left ventricular end diastolic pressure. Am J Cardiol 9: 237–247. 14474629 10.1016/0002-9149(62)90043-7

[22] Psaty BM, Manolio TA, Kuller LH, Kronmal RA, Cushman M, et al (1997) Incidence of and risk factors for atrial fibrillation in older adults. Circulation 96: 2455–2461. 9337224 10.1161/01.cir.96.7.2455

[23] Russo C, Jin Z, Homma S, Rundek T, Elkind MS, et al (2012) Left atrial minimum volume and reservoir function as correlates of left ventricular diastolic function: impact of left ventricular systolic function. Heart 98: 813–820. 22543839 10.1136/heartjnl-2011-301388 PMC3392716

[24] Guan Z, Zhang D, Huang R, Zhang F, Wang Q, et al (2010) Association of left atrial myocardial function with left ventricular diastolic dysfunction in subjects with preserved systolic function: a strain rate imaging study. Clin Cardiol 33: 643–649. 20960540 10.1002/clc.20784 PMC6653575

[25] Liu YY, Xie MX, Xu JF, Wang XF, Lv Q, et al (2011) Evaluation of left atrial function in patients with coronary artery disease by two-dimensional strain and strain rate imaging. Echocardiography 28: 1095–1103. 21967171 10.1111/j.1540-8175.2011.01513.x

[26] Dokainish H, Nguyen JS, Sengupta R, Pillai M, Alam M, et al (2010) Do additional echocardiographic variables increase the accuracy of E/e' for predicting left ventricular filling pressure in normal ejection fraction? An echocardiographic and invasive hemodynamic study. J Am Soc Echocardiogr 23: 156–161. 20152696 10.1016/j.echo.2009.11.015

[27] Pirat B, Khoury DS, Hartley CJ, Tiller L, Rao L, et al (2008) A novel feature-tracking echocardiographic method for the quantitation of regional myocardial function: validation in an animal model of ischemia-reperfusion. J Am Coll Cardiol 51: 651–659. 18261685 10.1016/j.jacc.2007.10.029 PMC3348770

[28] Kalay N, Celik A, Inanc T, Dogan A, Ozdogru I, et al (2011) Left Ventricular Strain and Strain Rate Echocardiography Analysis in Patients with Total and Subtotal Occlusion in the Infarct-Related Left Anterior Descending Artery. Echocardiography 28: 203–209. 21210836 10.1111/j.1540-8175.2010.01298.x

[29] Kimura K, Takenaka K, Pan X, Ebihara A, Uno K, et al.. (2011) Prediction of coronary artery stenosis using strain imaging diastolic index at rest in patients with preserved ejection fraction. J Cardiol. 10.1016/j.jjcc.2011.01.008 21388788

[30] Nesbitt GC, Mankad S, Oh JK (2009) Strain imaging in echocardiography: methods and clinical applications. Int J Cardiovasc Imaging 25 Suppl 19–22. 19145475 10.1007/s10554-008-9414-1

[31] Min PK, Ha JW, Jung JH, Choi EY, Choi D, et al (2007) Incremental value of measuring the time difference between onset of mitral inflow and onset of early diastolic mitral annulus velocity for the evaluation of left ventricular diastolic pressures in patients with normal systolic function and an indeterminate E/E'. Am J Cardiol 100: 326–330. 17631092 10.1016/j.amjcard.2007.02.102

[32] Paulus WJ (2010) Novel strategies in diastolic heart failure. Heart 96: 1147–1153. 20610461 10.1136/hrt.2009.169052

[33] Stolker JM, Rich MW (2010) Clinical utility of B-type natriuretic peptide for estimating left ventricular filling pressures in unselected elderly patients undergoing diagnostic coronary angiography. J Invasive Cardiol 22: 107–112. 20197576

[34] Sigwart U, Grbic M, Goy JJ, Kappenberger L (1990) Left atrial function in acute transient left ventricular ischemia produced during percutaneous transluminal coronary angioplasty of the left anterior descending coronary artery. Am J Cardiol 65: 282–286. 2301255 10.1016/0002-9149(90)90288-c

[35] Stewart JT, Grbic M, Sigwart U (1992) Left atrial and left ventricular diastolic function during acute myocardial ischaemia. Br Heart J 68: 377–381. 1449920 10.1136/hrt.68.10.377 PMC1025136

[36] Amundsen BH, Helle-Valle T, Edvardsen T, Torp H, Crosby J, et al (2006) Noninvasive myocardial strain measurement by speckle tracking echocardiography: validation against sonomicrometry and tagged magnetic resonance imaging. J Am Coll Cardiol 47: 789–793. 16487846 10.1016/j.jacc.2005.10.040

[37] Stefanadis C, Dernellis J, Tsiamis E, Toutouzas P (1999) Effects of pacing-induced and balloon coronary occlusion ischemia on left atrial function in patients with coronary artery disease. J Am Coll Cardiol 33: 687–696. 10080469 10.1016/s0735-1097(98)00623-8

[38] Her AY, Kim JY, Kim YH, Choi EY, Min PK, et al.. (2012) Left Atrial Strain Assessed by Speckle Tracking Imaging Is Related to New-Onset Atrial Fibrillation After Coronary Artery Bypass Grafting. Can J Cardiol. 10.1016/j.cjca.2012.06.006 22902158

[39] Motoki H, Dahiya A, Bhargava M, Wazni OM, Saliba WI, et al (2012) Assessment of left atrial mechanics in patients with atrial fibrillation: comparison between two-dimensional speckle-based strain and velocity vector imaging. J Am Soc Echocardiogr 25: 428–435. 22265458 10.1016/j.echo.2011.12.020

